# Regulatory mechanism of miR-20a-5p expression in Cancer

**DOI:** 10.1038/s41420-022-01005-5

**Published:** 2022-05-16

**Authors:** Wei Huang, Xiaoyue Wu, Shuaixi Xiang, Mingxin Qiao, Xiao Cen, Xuefeng Pan, Xinqi Huang, Zhihe Zhao

**Affiliations:** State Key Laboratory of Oral Diseases & National Clinical Research Center for Oral Diseases, West China Hospital of Stomatology, Sichuan University, Chengdu, 610041 P. R. China

**Keywords:** RNA metabolism, Cancer

## Abstract

MicroRNAs(miRNAs) are non-coding single-stranded RNA molecules encoded by endogenous genes with a length of about 22 nucleotides. The dysregulation of miRNAs has been proven to be one of the vital causes of cancer, which makes them a biomarker for cancer diagnosis and prognosis. Compared with surgery and chemotherapy, nucleic acid therapy targeting specific miRNAs is a promising candidate for cancer treatment. miR-20a-5p plays an anticancer role in high-incidence human cancers such as cervical cancer, breast cancer and leukemia, which is of great importance in the diagnosis of cancers. The up-regulation and down-regulation of miR-20a-5p offers a possible breakthrough for the treatment of cancers. In this paper, we aim to investigate the functional significance of miR-20a-5p in different cancers, reviewing the expression differences of miR-20a-5p in cancer, while systematically summarizing the changes of circRNA-miR-20a-5p networks, and probe how it promotes messenger RNA (mRNA) degradation or inhibits mRNA translation to regulate downstream gene expression. We’ve also summarized the biogenesis mechanism of miRNAs, and emphasized its role in cell proliferation, cell apoptosis and cell migration. On this basis, we believe that miR-20a-5p is a promising and effective marker for cancer diagnosis, prognosis and treatment.

## Facts

- MiRNAs are involved in the proliferation, movement and metastatic activity of oncocytes.
- miR-20a-5p serves as a cancer diagnosis and prognosis marker in breast cancer, liver cancer, leukemia, etc.
- MiR-20a-5p promotes or inhibits cancer by targeting downstream signal channels.
- Circrna-miR-20a-5p network plays a regulatory role in cancer through the sponge mechanism.
- MiR-20a-5p provides a new breakthrough for the development of targeted anticancer drugs.

## Open question

- What is the downstream regulation mechanism of miR-20a-5p in cancer?
- How can miR-20a-5p inhabit the proliferation and movements of cancers?
- What is the relationship between circular RNA and cancers? How does it interact with miR-20a-5p?

## Introduction

Cancer refers to an abnormal growth of cells that can invade other parts of the body and is caused by an aberration in the mechanism controlling cell division and proliferation [1], and it’s the 2^nd^ leading cause of mortality across the globe, causing nearly 1/6 of the total deaths [2]. Common carcinogenic factors include obesity [3], biological infection [4], free radiation [5], environmental pollutions [6], etc. It is important to note that carcinogenic factors usually function by altering the genetic material in cells, such as DNA damage related to cell proliferation, abnormal transcription and modification of related proteins, oncogene activation caused by viral infection [7].

MicroRNAs (miRNAs) are a class of endogenous non-encoded RNAs with a length of approximately 22 nT, which act as posttranscriptional regulators of genetic expression, most of which have high sequence conservative, expression timing and tissue specificity [8]. MiRNAs can function as cancer suppressor and cancer promoter, and specific miRNAs overexpression and interference can be leveraged to study the role of miRNAs in the occurrence and progression of tumor. In addition, miRNAs expression in normal and cancer tissues is significantly altered, which makes miRNAs a potential biomarker for tumor diagnosis [9]. Due to the specific role of miRNAs in cancer, some specific miRNAs can be designed to target cancer therapy.

As a component of the mir-17–92 cluster, miR-20a-5p is confirmed to be closely associated with cancer in many fields [10]. Its mechanisms include the regulation of continuous proliferation signals, tax-evading growth inhibition, invasive and metastatic activation, replication immortality, angiogenic ability, resistance to cellular death and avoidance of immunity damage [11].

Numerous studies have focused on the effect of miR-20a-5p on carcinomas. Overexpression or downregulation of miR-20a-5p may affect the downstream signaling pathway, such as PI3K-Akt [12], MAPK [13], and TGF-β signal paths [14]. It further affects the expression of related proteins, thus forming the function of promoting or inhibiting cancer. Some studies focused on the upstream signal expressing of miR-20a-5p and discovered that the circRNA-miRNA network was closely associated with cancer, suggesting that circRNA-miRNA network may be a promising anticancer drug target [15].

In the present paper, our team reviewed the effect of miR-20a-5p on common human cancers, discussing the influence of its upstream and downstream signaling pathways, and emphasized its possible carcinogenic mechanism in order to offer novel illuminations for the treatment of human cancers.

## Overview of miR-20a-5p

### Roles and mechanisms of miRNAs

**Fig. 1:**
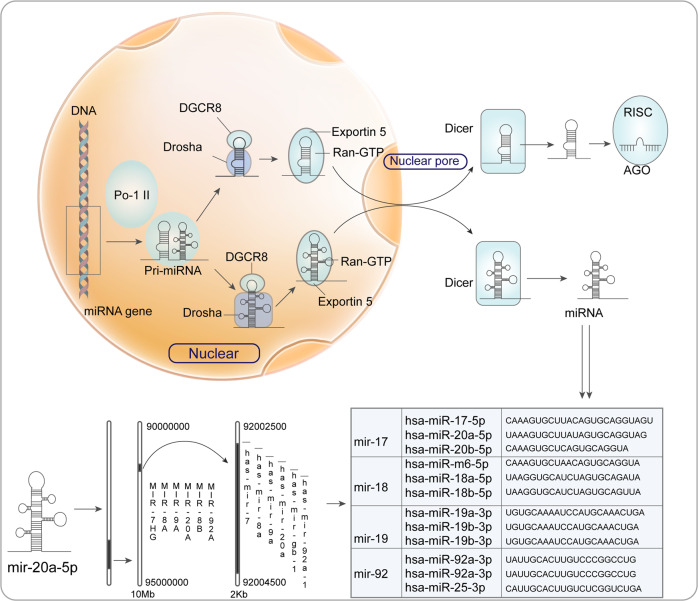
The biogenesis of miR-20a-5p. miR-20a-5p is first transcribed by RNA polymerase II (Pol II) and pri-miRNA is obtained. Pri-miRNA is cleaved continuously to obtain multiple different miRNAs. In the nucleus, Drosha cuts pri-miRNA, which is exported from the nucleus by binding to RAN-GTP and Exportin 5. In the cytoplasm, Dicer endoribonuclease cleaves the ring structure to produce mature miR-20a-5p. miR-20a-5p is a family of miR-17, located on human chromosome 13. The sequence and nucleotide sequence of miR-20a-5p gene are shown in the figure.

miRNAs are a class of single-stranded ncRNA molecules, which are around 22 nucleotides in length, encoded by endogenetic genes [16], and they regulate genetic expression by identifying homologous sequences and interfering transcription, translation or apparent genetic process [8] (Fig. 1). The miRNAs gene is transcribed into pri-RNA by RNA Pol II in the nucleus. Then pri-miRNAs are subjected to cleavage by RNase III Drosha into approximately 70nt in length, an exportin-like structure of pre-miRNAs, which is afterwards transferred to the cytoplasm by exportin-5 transporter. After the action of RNase Dicer-like 1 (DCL1), short strand RNA of 21~ 23nt is generated, and mature miRNAs are further formed [17]. Mature miRNAs bind to Argonaute (AGO) protein and bind with their assistance to RNA-induced silencing complex (RISC) [18]. After binding to the targeted mRNA, the 5’ -end of the microRNA complements mRNA3’ -UTR, resulting in decomposition of the mRNA or suppression of its translational process and the regulation of gene expression [8]. When the miRNAs are completely or almost completely complementary to the target mRNA, the target mRNA will degrade [19]. When miRNAs and target mRNA are not completely complementary, the translation process is negatively regulated and protein translation is blocked [20].

In clinical practices, miRNAs have been proven to be closely related to cardiovascular diseases, cancer and respiratory diseases. Researches have revealed that the abnormal highly-expressed oncogenic miRNAs (OncomiRs) directly bind to multiple tumor suppressor genes and down-regulates the expression of these mRNAs, thus leading to the proliferation, movement and metastatic activity of oncocytes [21]. In respiratory diseases, miRNAs can also be packaged as exosomes or micro vesicles and excreted to the exocellular milieu, involving a variety of biofluids, thus enabling long-distance cell-to-cell communication [22].

### miR-20a-5p serves as a cancer diagnosis and prognosis marker

As one of the miRNAs researched in depth, miR-20a, a component of the miR-17–92 cluster, is a conserved gene belonging to vertebrates [23]. There is a close association between the components of miR-17/92 cluster, and miR-17 and miR-20a can jointly regulate the expression of E2F1 and target 30 UTRs of E2F2 and E2F3 [24]. In terms of TGF- B signal transmission, miR-17 and miR-20a can also straightly target TGFBRII [25].

miR-20a-5p is confirmed to be closely related to respiratory diseases, cardiovascular diseases, and cancer in a large number of in vitro and in vivo experiments [26, 27]. This is usually due to the over-expression or downregulation of upstream processing machinery components leading to abnormal biosynthesis of miR-20a-5p, which further modifies the overall miRNA protein expression [10]. Heterozygosis XPO5-inactivation mutations triggering the damage of pre-miRNA exportation from nuclei to cytoplasm are discovered in endometrium, colon, and stomach cancers with micro-satellite unsteadiness [28].

In some diseases, the characteristic expressing of miR-20a-5p could be utilized as a basis for disease diagnosis. miR-20a-5p is overexpressed in various tumors, such as HNSCC [29], breast cancer [30], ovary carcinoma [31], etc. Therefore, miR-20a-5p can be used as a hallmark for cancers. miR-20a-5p is also significantly enriched in cancer-related pathways, which contributes to the initiation of breast cancer [32], cervical cancer [33], leukemia [34] and other cancers. Numerous studies have explored the regulatory role of miR-20a-5p in cancer as well as the upstream or downstream channels that may affect its expression [35]. miR-20a-5p can be used as a clinical diagnostic indicator of some cancers. In mammary carcinomas, the expressing of miR-20a-5p exhibits an upward trend and can be used as a detection indicator [36]. It can also be leveraged as a non-invasive biomarker for different hematological malignancies, such as multiple myeloma, leukemia and lymphoma [10].

## miR-20a-5p-mRNA net in cancers

### Breast cancer

Breast cancer is a non-controllable proliferation of mammary gland epithelial cells under the influence of a variety of carcinogenic factors [37]. The prevalence of mammary carcinomas is 24.2 percent worldwide [38], ranking first among female cancers. Signs of breast cancer include lumps, changes in the shape of the breast, sunken skin, and patients with distal metastasis may have bone pain, enlarged lymph nodes, dyspnea, or jaundice [39, 40]. Chemotherapy is a common treatment for breast cancer, but it is prone to drug resistance and severe adverse reactions [41]. Molecular targeted therapy for breast cancer has made new progress. Multiple adenosine diphosphate ribose polymerase (PARP) inhibitors can target TNBC and show an ORR of 82.0% in patients with PALB2 embryogenic mutations [42]. Sacituzumab Govitecan (SG) is also used for TNBC. Clinical results showed that THE ORR of SG in patients with metastatic TNBC was 33.3%, and the median PFS time was 5.5 months [43].

Numerous researches have revealed that miR-20a-5p is vital for the development and prognosis of breast cancer through the targeted regulation of genes such as SPRy4-IT1 [44], SRCIN1 [45] and PANDAR [46], and have also provided a breakthrough for drug design and synthesis of breast cancer. Hong et al. discovered that miR-20a-5p could be significantly up-regulated in TNBC, which could be used as one of the reference standards for the prediction model of postoperative recurrence in TNBC patients [47]. Bai et al. indicated that the highly-expressed miR-20a-5p could remarkably reduce the mRNA and protein content of RUNX3 and the immediate downstream target genes Bim and P21, which could promote the migration and invasion of TNBC cells [48]. Guo et al. found that miR-20a-5p from exosomes derived from mammary carcinoma cells promoted the proliferative and differentiative abilities of osteoclasts via targeting SRCIN1, offering a reasonable basis for targeting exosomes or miR-20a-5p to intervene in the development of mammary carcinoma [49].

### Cervical cancer

Cervical cancer is induced by long-term chronic infection of cervical epithelial cells by human papillomavirus, resulting in poor metaplasia of epithelial cells [50]. Cervical cancer accounts for 5% of all new cancer cases worldwide, with about 500,000 new cases each year [51]. The symptoms of advanced cervical cancer vary, depending on which organ has been invaded [51, 52]. Cervical cancer vaccines, such as Gradasil MSD and Cervirax GSK, can effectively prevent nearly 100% human papillomavirus type 16 and 18 infection [52, 53]. But it does not prevent the remaining 30 percent or so of cancers caused by infections with other viruses. Therapeutic approaches targeting miR-20a-5p are still promising and of great research value.

The diagnosis of cervical cancer includes the analysis of changes in serum levels of specific miRNAs and the detection of abnormal hypermethylation of miRNAs [33]. miR-20a-5p is confirmed to be up-regulated in mammary carcinoma, and as an early biomarker, it exhibits higher sensitivity and specificity compared with squamous cell carcinoma (SCC), cancer antigen (CA)-125, and CA19-9 [33]. miRNAs can affect HPV DNA replication, which provides more clues to our understanding of the life cycle of HPV and the mechanistic basis of HPV-induced tumorigenesis. Qin et al. found that miR-20a-5p promoted the progression of cervix carcinoma via targeting RUNX3. DR5 is an important promoter of NK cell activation and a downstream target of RUNX3. Therefore, miR-20a-5p promotes the cytotoxicity activity of NK cells via modulating RUNX3/DR5 axis, thus inhibiting the development of cervical cancer [54].

### Endometrial cancer

Endometrium carcinoma is a series of epithelium malignancies occurring in the endometrial region, most commonly occurring in peri-menopausal and post-menopausal females [55]. Endometrium carcinoma is one of the most commonly seen cancers of female reproduction systems [56], with a few patients having vagina bleeds or serous sex secretion [57]. Surgeries are still the treatment of choice for endometrial cancer, but most of which are so damaging to the uterus that patients are unable to conceive. Chemotherapy and progesterone therapy can be used for the early treatment [58]. Current potential molecular targeted drug therapies for endometrial cancer include PI3K/Akt/mTOR inhibitors [59], FGFR [60], EGFR [61], histone deacetylase inhibitors [62], etc., with Bevacizumab widely used in clinical treatment [63]. However, the above-mentioned treatment methods have large side effects and irreversible damage to human body. Mirna-targeted drugs are still a research hotspot.

miR-20a-5p is confirmed to be remarkably down-regulated in endometrial carcinoma. He et al. reported that miR-20a-5p might inhibit cancer via targeting Jak1 expression [64]. Huang et al. discovered that miR-20a-5p suppressed the expressing of STAT3 through direct interaction. The down-regulation of STAT3 inhibits the invasion of EMT and EC cells. In addition, the loss of STAT3 will weaken the invasion of EMT and endometrial cancer induced by the down-regulation of miR-20a-5p, and is vital for endometrial cancer metastasis and malignant transformation [65]. Corpulence and the factors related to metabolism syndromes, such as diabetic diseases and PSO, are risky factors for the progression of endometrium carcinoma. miR-20a-5p is a positive modulator of the differentiative activity of lipocytes and adipogenesis in 3T3-L1 [66]. Zhu et al. proposed that siRNA-mediated Klf3 silencing reproduced the enhanced lipogenesis triggered by the over-expression of miR-20a-5p, while the reinforced Klf3 expressing attenuated the role of miR-20a-5p. It is speculated that miR-20a-5p facilitates adipocyte differentiation from bone marrow derived matrix cells by targeting and negatively regulating Klf3 in the early stage of adipogenesis [67].

### Acute myeloid leukemia

Acute myeloid leukemia (AML) is a cancer caused by the excessive proliferation of blood cells in the bone marrow [68], with an annual incidence of 1.62 per 100,000 people [69]. It is characterized by the rapid growth of substantial abnormal cells in the bone marrow and blood, which interferes with hematogenesis. The relevant symptoms might involve tiredness, dyspnea and elevated infection risks [70]. Acute myelogenous leukemia is usually treated with chemotherapy to achieve remission, and patients may undergo further chemotherapy, radiation therapy, or bone marrow transplantation after remission [71]. At present, a large number of researchers are developing targeted drugs for leukemia, which target CD33, CD22, FLT3, IDH2, IDH1, BCR-ABL and so on [72]. Some of the new drugs have been put into clinical use or entered the stage of pending approval.

Numerous studies have shown that miR-20a-5p is remarkably reduced in the bone marrow of AML sufferers, and its reduced expressing is related to the risk status and poor survival prognostic results of AML sufferers, suggesting that this is a possible drug target [34]. Bao et al. found that PPP6C, as a targeted gene of miR-20a-5p, is modulated in AML cells in a negative way and negatively affects G1/S transition and apoptosis [73]. Ping et al. found that knocking down CIRC_0009910 suppressed AML cellular proliferative ability and triggered programmed cell death via sponging [74]. Liu et al. revealed that curcumin would inhibit the proliferation and migration of AML cells and block cell cycle progression by regulating HOTAIR/ miR-20a-5p /WT1 axis [75].

### Liver cancer

Liver cancer is a malignant tumor occurring in or from the liver [76]. The most common type of liver cancer is hepatocellular carcinoma (HCC), which accounts for 80% of all cases, followed by cholangiocarcinoma [77]. The commonly seen clinic features are hepatic pains, abdomen distension, bad appetite, tiredness and upper abdomen mass, etc. Certain sufferers would have low fever, icterus, and acute abdominal diseases posterior to the rupture of hepatic carcinoma [76]. At present, the treatment mode of liver cancer has changed from single local treatment to multi-disciplinary comprehensive treatment including surgery, ablation, intervention, targeting, immunotherapy and so on [78]. Compared with local treatment, remarkable advancements have been achieved in terms of medicinal therapies for liver cancer. Drugs commonly target fibrocell growth factor receptor 4 (FGFR4) [79], PD-1 antibody [80], TGFβ [81], and A3 adenosine receptor (A3AR) [82], etc.

Among the above targets, miR-20a-5p shows its unique function as providing a possible low-risk treatment. Wen et al. discovered that miR-20a-5p was overexpressed in HCC and could be used as a preclinical biomarker for HCC [83]. Fu et al. found that the downregulation of miR-20a-5p led to TGFBR2 activation of TGF-β signaling pathway, followed by the activation of macrophages and extracellular matrix (ECM) by hepatic stellate cells (HSC), promoting the development of hepatic fibrosis [14]. Chen et al. proposed that miR-20a-5p overexpression promoted HCC cell proliferation and migration by reducing RUNX3 translation [84].

### Osteosarcoma

Osteosarcoma (OS) is one of the most common bone malignancies. Its rapid tumor growth is due to the formation of tumor osteoid and bone tissue directly or indirectly through the cartilaginous stage [85]. The prominent symptom of osteosarcoma is pain at the tumor site caused by infiltration and dissolution of bone cortex by tumor tissue [86]. At present, the accepted treatment mode of osteosarcoma is the constitution of neoadjuvant chemotherapy before surgery, tumor resection and adjuvant chemotherapy after surgery [82]. First-line chemotherapy drugs are mainly adriamycin [87], methotrexate [88], cisplatin [89] and ifosfamide [87], but not all patients can exhibit good response, and some patients even have significant drug resistance.

Studies have found that miR-20a-5p, as one of the hallmarks of osteosarcoma, is significantly reduced in the serum expression of OS patients, which has the possibility of drug targeting [90]. Zhao et al. found that miR-20a-5p targeting SDC2 triggered the inhibition of OS chemotherapy resistance and proposed that miR-20a-5p /SDC2 axis might become an underlying diagnosis marker and treatment target for OS sufferers [91].

### Other malignancies

In addition to the above-mentioned cancers, miR-20a-5p also exerts a regulatory effect on many other cancers (Table 1).

**Table 1:** Regulation of miR-20a-5p in cancer.

Disease	Expression of miR-20a-5p	Target	Expression	Distribution	Cell source	Function	Experiment	Animal model	Reference
Breast cancer	↓	HMGA2	↓	Cytoplasm	Human and mouse	BC cells progression was medicated by LncRNA HOTAIR via miR-20a-5p/HMGA2, further influencing cell growth, metastasis and apoptosis	In vitro	-	[30]
	↑	RUNX3	↓	Nucleus	Human and mouse	miR-20a-5p targeted RUNX3, thus facilitating the proliferation and migration of TNBC cells	In vitro and in vivo	Nude mice/8	[48]
	↑	SRCIN1	↓	Exosome	Human	miR-20a-5p transferred from breast cancer cell-derived exosomes promotes the proliferation and differentiation of osteoclasts by targeting SRCIN1	In vitro	-	[49]
Cervical cancer	↑	RUNX3	↓	Nucleus	Human and mouse	LINC00657 suppressed cervical cancer progression via inducing miR-20a-5p/RUNX/DR5 mediated NK cell tolerance	In vitro and in vivo	Nude mice/12	[54]
Endometrial Cancer	↓	Jak1	↓	Cytoplasm	Human and mouse	miR-20a-5p acted as a tumor suppressor in EC partly via decreasing Jak1 expression	In vitro	-	[64]
	↓	STAT3	↓	Cytoplasm	Human and mouse	miR-20a-5p inhibited EMT and invasion of EC cells by targeting STAT3	In vitro	-	[65]
Leukemia	↓	PPP6C	↓	Nucleus	Human and mouse	miR-20a-5p was downregulated in AML through negatively regulating PPP6C expression	In vitro and in vivo	Nude mice/10	[73]
	↓	SOX4	↓	Cytoplasm	Human and mouse	circ PRKCI contributed to the malignant progression of T-cell acute lymphoblastic leukemia by miR-20a-5p/SOX4 axis	In vitro	-	[103]
Liver cancer	↓	TGFBR2	↓	Cytoplasm	Human and mouse	The downregulation of miR-20a-5p in liver fibrosis resulted in TGFBR2-activated TGF-beta signaling pathway, revealing the critical role of miR-20a-5p in liver fibrosis development	In vitro	-	[14]
	↑	RUNX3	↓	Cytoplasm	Human and mouse	miR-20a-5p overexpression contributed to HCC cell proliferation and migration through reducing the translation of RUNX3	In vitro	-	[84]
	↓	ERBB3	↓	Nucleus	Human and mouse	miR-20a-5p could suppress the metastasis of hepatocellular carcinoma through its target gene ERBB3	In vitro and in vivo	Nude mice/30	[105]
	↑	Smad4	↓	Cytoplasm	Human and mouse	miR-20a-5p promoted the invasion and metastasis ability by suppressing Smad4 expression in CRC cells	In vitro and in vivo	Nude mice/5	[106]
Osteosarcoma	↑	SDC2	↓	Cytoplasm	Human and mouse	miR-20a-5p can regulate OS multi-drug resistance through its direct target gene SDC2	In vitro and in vivo	Nude mice/8	[91]
	↑	KIF26B	↓	Cytoplasm	Human and mouse	miR-20a-5p can regulate OS multi-drug resistance through its direct target gene KIF26B	In vitro and in vivo	Nude mice/12	[107]
Non-small cell lung cancer	↓	RRM2	↓	Cytoplasm	Human and mouse	miR-20a-5p suppressed NSCLC growth by inhibiting RRM2-mediated signaling pathway	In vitro and in vivo	Nude mice/40	[93]
Head and neck squamous cell carcinoma	↑	TNFRSF21	↓	Cytoplasm	Human and mouse	miR-20a-5p functioned as an oncogene in HNSCC by downregulating TNFRSF21	In vitro	-	[108]
Neuroblastoma	↓	ATG7	↓	Cytoplasm	Human and mouse	miR-20a-5p was downregulated while ATG7 was upregulated in NB progression	In vitro	-	[92]

miR-20a-5p is often overexpressed in HNSCC sufferers. Wu et al. found that the up-regulation of miR-20a-5p promoted the proliferative and invasive abilities of HNSCC cells via targeting TNFRSF21 [29]. Yu et al. discovered that miR-20a-5p suppressed cellular proliferative ability and promoted programmed cell death via modulating ATG7 in a negative way, thus inhibiting the autophagy of SH-SY5Y cells, providing a new idea for Neuroblastoma treatment [92]. Studies have shown that miR-20a-5p inhibits tumorous angiogenic activity in NSCLC via the RRM2-mediation PI3K/Akt signal path, which may be an effective molecular target for the treatment of NSCLC [93].

**Fig. 2:**
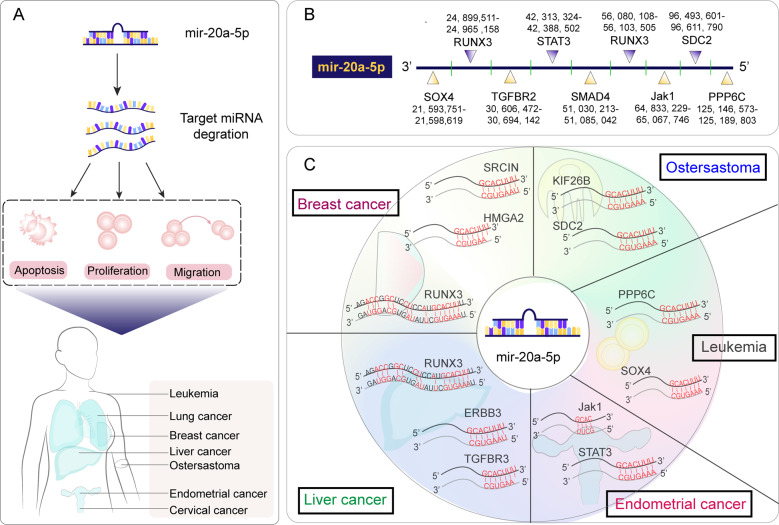
miR-20a-5p expression regulations in cancers. **A** During the development of cancer, the expression level of miR-20a-5p changes, which promotes apoptosis, proliferation and migration of cancer cells, and ultimately leads to the development of various human cancers. **B** Binding sites of miR-20a-5p to targeted gene. **C** miR-20a-5p promotes the occurrence of cancer by inhibiting or promoting targeted genes.

Above all, miR-20a-5p is confirmed to be overexpressed or suppressed in many cancers (Fig. 2), and more mysteries remain unknown in terms of its upstream or downstream signaling pathways. However, there is no doubt that miR-20a-5p is vital for cancer, which is of great significance as a biomarker and provides new ideas for the design of targeted drugs.

## circRNA-miR-20a-5p network in cancers

### Overview of circRNA

circRNA is a class of ncRNA molecules with a closed ring morphology, with no A 5’ cap structure and 3’poly (A) structures [94]. circRNA is primarily located in the cytoplasm or stored in exosomes. It isn’t influenced by RNA exonuclease, with a more stable expression, and it’s hard to be decomposed, proven to exist widely in various eukaryotes [95]. Most circRNAs are cyclized by exons, while some circRNAs are lasso structures (lariat) formed by cyclized introns. At the same time, circRNA contains a large number of miRNAs response elements (MREs), which is capable of forming the catalysis core of RISC with AGO protein, ultimately leading to circRNA degradation [96].

circRNA cyclization can be divided into intron cyclization and exon cyclization. Currently, the mainstream cyclization mechanisms include the following three types. First, the clipping of cable tail depends on the shear body. On the mRNA precursor, the 5’ donor site downstream of the exon is connected to the 3’ receptor site upstream by continuous assembly of the small nuclear ribosome protein, and the loop is inserted to form circRNA, and then the circRNA is formed by shearing [95]. Second, cis-acting elements promote circRNA formation. The introns on the two sides of some circRNA exons have inverse complement sequences. RNA double-stranded bodies are formed side by side at the shearing site, and then two diverse circRNAs with and with no introns are produced through variable shearing. Introns inside and on both sides of the exon are capable of competing for RNA pairing and eventually generate diverse kinds of circRNAs by variable shearing [97]. Thirdly, RBPs modulate the forming of circRNAs. circRNA formation is promoted by binding RBPs to introns on exon flanks [98].

### The mechanism and function of circRNA-miRNA network

**Fig. 3:**
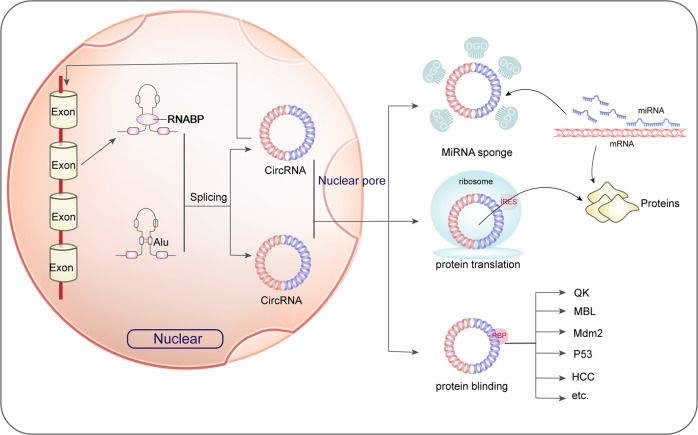
Biogenesis and function of circRNA-miRNA network. circRNA is formed by pre-mrna through back-splicing. Generated circRNA usually has three functions: adsorbing miRNA as sponge, encoding and translating into short peptides, and binding functional proteins to regulate its intracellular functions.

circRNA is abundant in miRNAs binding spots, being a miRNAs sponge and avoiding the interaction between miRNAs and mRNAs in the 3’ untranslated area, and then it regulates the expressing of downstream targeted genes of miRNAs in an indirect way [99] (Fig. 3).

Multiple experiments have predicted and confirmed that circRNA, as an endogenous competitive RNA, can compete for miRNAs through MREs. For example, Cdr1as contains about 70 conserved miR-7 binding sites and a miR-671 binding site [100]. Due to their different complementary ways with Cdr1as sequences, miR-671 can trigger the degradation of Cdr1as and indirectly affect the level of miR-7 [101]. Notably, abnormal circRNAs in cancer have also been identified as chromosomal translocations of proto-oncogene products. circRNA is associated with multiple cancers in vivo. In terms of gene expression, the acute and chronic targeting of Cdr1as may have positive significance for treatment [102].

Based on the sponge regulation, the circRNA-miRNA network is vital for the regulation of cancer, and the abnormal expression of upstream circRNA will affect the expression of downstream miRNAs and even mRNA. Therefore, the study of circrNA-miRNA net is of great significance for the further understanding of human cancer.

### Roles of circRNA-miR-20a-5p net in cancers

A large number of binding sites exist between circRNA and miR-20a-5p, allowing circRNA to competitively bind to miR-20a-5p and playing the role of sponge regulation. As a consequence, the function of miR-20a-5p is inhibited by circRNA which indirectly do damages to the regulatory effect of miR-20a-5p on downstream target gene. Multiple studies have discussed in depth the mechanism of circRNA-miR-20a-5p network in diseases, and have found that circRNA-miR-20a-5p axis played a vital role in the process of cancers (Table 2).

**Table 2:** circRNA-miR-20a-5p network in regulating cancers.

circRNA	circRNA expression	Distribution	Identification of circular RNA	Cell source	Verification of circular structure	Binding verification	Effect on osteoporosis	Reference
Circ_0009910	↑	Cytoplasm	Microarray analyses	Human	-	Luciferase reporter assays, RIP, RNA pull-down assays	The knockdown of Circ_0009910 suppressed acute myeloid leukemia cell growth	[74]
CircPRKCI	↑	-	Previous report	Human	RNase R	Luciferase reporter assays	CircPRKCI promoted the malignant progression of T-cell acute lymphoblastic leukemia	[103]
Hsa_circ_0107593	↓	-	Previous report	Human	RNase R	Luciferase reporter assays	Luciferase reporter assays hindered the processes of cervical cancer	[104]

In Lei Ping’s study, it was found that circ_0009910 expression significantly upregulated while miR-20a-5p expression declined in the acute myeloid leukemia (AML) patients compared with iron-deficiency anemia patients. Knocking down circ_0009910 suppressed the growth of AML5 cells through the regulation of miR-20a-5p, and luciferase reporter assay, RNA RIP assay and RNA pull down assay also confirmed the particular binding relationship between circ_0009910 and miR-20a-5p [74]. SOX4 is considered as one of the oncogene which is often found in cancers, it was observed that SOX4 was involved in the process of T cell leukemia and SOX4 could regulate the expression of CXCL13 in human T helper cells. In Yan Zheng et al. study, SOX4 expression decreased in T-cell acute lymphoblastic leukemia(T-ALL), and knocking down SOX4 leads to a decreasing number of T-ALL cells. Meanwhile, circPRKCI was found to have a positive correlation with SOX4 while miR-20a-5p was negatively correlated with SOX4. Knockdown of circPRKCI suppressed the survival of T-ALL cells via sponging miR-20a-5p, and miR-20a-5p could subsequently apply inhibitory effect on SOX4. Therefore, downregulation of circPRKCI could repress the malignant progression of T-ALL through miR-20a-5p/SOX4 signaling pathway [103]. In cervical cancer, miR-20a-5p expression increased during the process of cervical cancer and overexpression of miR-20a-5p would aggravate cervical cancer. Luciferase reporter assay showed hsa-circ-0107593 could bind with hsa-miR-20a-5p where hsa-circ-0107593 acted as a sponge of hsa-circ-0107593 to inhibit the process of cervical cancer [104]. Taken together, it can be seen that miR-20a-5p could as a pivot which bridges upstream circRNA and downstream mRNA to regulate the processes of cancers.

Research on circRNA-miRNA network continues to advance. With the deepening of scholars’ understanding of circRNA-miRNA networks, we find that circRNA-targeted regulation is a new pathway for cancer treatment, providing new possibilities for the research and development of related drugs.

## Conclusions

Recently, massive studies have revealed the mechanism of miRNAs in cancer in great detail. The overexpression or downregulation of miRNAs can regulate the downstream signaling channels or directly affect the synthesis of related proteins, which are closely associated with cancer. Its upstream channels, especially circRNA, also exhibit strong correlation with miRNAs. The changes of circRNA, miRNAs, and mRNA extensively affect many aspects of tumorigenesis, and regulating their expression can interfere with their cancer-promoting function, providing opportunities for cancer treatment and intervention.

As a newly discovered class of miRNA, although several hypotheses have been proposed for its role in tumors, the biology function and mechanism of miR-20a-5p are still elusive, which requires further in-depth analysis of its mechanism of action in tumors as well as correct treatment methods. In addition, given that circRNA plays a key role in tumor progression through sponging miRNAs, future studies should better elucidate its possible application in tumor diagnosis and treatment.

In this review, it was found that miR-20a-5p could play a vital role on the cell proliferation, invasion and metastasis in various cancers. Accumulated evidences have indicated that miR-20a-5p is widely expressed in a variety of cells, it can not only interact with some circRNAs, but also miR-20a-5p is associated with lots of downstream gene which regulates the processes of malignant tumor. Besides, miR-20a-5p is also involved in some pathways like PI3K/AKT signaling pathway. As a biomarker, the miR-20a-5p expression changed obviously in different type of cancers which makes it a potential diagnostic and prognostic value. However, in order to make miR-20a-5p as a diagnosis or prognosis biomarker, more cases should be studied to clarify the threshold for different cancers. And the role of miR-20a-5p in cancer and its underlying mechanism need further study. We sincerely wish to further study the carcinogenic mechanism of miR-20a-5p and provide a promising new direction for cancer treatment.
